# Intralesional antibiotic injection using 41G needle for the management of subretinal abscess in endogenous endophthalmitis

**DOI:** 10.1186/s40942-016-0043-x

**Published:** 2016-08-01

**Authors:** Pradeep Venkatesh, Shreys Temkar, Koushik Tripathy, Rohan Chawla

**Affiliations:** Dr. Rajendra Prasad Centre for Ophthalmic Sciences, All India Institute of Medical Sciences, E-104, AIIMS Campus, Ansari Nagar, New Delhi, 110029 India

## Abstract

**Background:**

Presence of subretinal abscess in endogenous endophthalmitis indicates a more severe form of infection. Available reports indicate variable response to standard treatment with systemic, intravitreal pharmacotherapy and vitreous surgery. There are no reports citing the possible role of intralesional antibiotic therapy in managing subretinal abscess.

**Case presentation:**

A 30 year old lady presented with features of endogenous endophthalmitis and subretinal abscess. Presenting vision was finger counting close to face. Despite prompt initiation of systemic antibiotics and intravitreal injection, no response was noted. 25G pars plana vitrectomy was performed along with injection of vancomycin directly into the subretinal abscess, using a 41G needle. Postoperative course was uneventful and the abscess showed signs of rapid resolution. Visual acuity improved to 6/24.

**Conclusion:**

Intralesional pharmacotherapy may be safe and effective in the treatment of subretinal abscess associated with endogenous endophthalmitis.

## Background

Endogenous endophthalmitis involves suppurative inflammation of the inner ocular coats by an infective focus arising from a systemic source. It accounts for 2–6 % of all endophthalmitis [1]. Subretinal abscess is an uncommon presentation and accounts for approximately 5 % of all endogenous endophthalmitis [2, 3]. It represents a severe form of the disease and requires appropriate systemic and local treatment [4, 5]. Various treatment modalities have been described but no standard management guidelines are available. We describe herein management of subretinal abscess by intralesional antibiotic injection using a 41G cannula. To the best of our knowledge such an approach for managing subretinal abscess has not been described earlier in published literature.

## Case presentation

A 30-year-old female patient presented with complaints of sudden onset painful and rapidly progressive diminution of vision in the left eye of 2 days duration. She gave a history of being administered intravenous drugs multiple times for her episodes of headache over the past 6 months. There was no history of trauma or ocular surgery. There was no history of any systemic illnesses.

On examination, best corrected visual acuity (BCVA) was 6/6 in the right eye and finger counting close to face with accurate projection of rays in the left eye. Intraocular pressure was 13 mmHg in the right eye and 10 mmHg in the left eye. On slit lamp biomicroscopy, there was mild ciliary congestion, anterior chamber cells of 4+ with a hypopyon of approximately 1 mm in the left eye. On indirect ophthalmoscopy, there was evidence of 3–4+ vitritis. A solitary elevated yellowish lesion could be hazily seen in the superotemporal quadrant giving suspicion of an abscess. Combined vector ultrasound B-scan confirmed the presence of a subretinal abscess associated with vitreous exudates. There was no evidence of retinal detachment or choroidal detachment.

On systemic examination she had evidence of cellulitis of the left lower limb. Blood cultures (aerobic and anaerobic bacterial and fungal) were sent before initiating intravenous antibiotics. Results of all other relevant investigations were normal.

**Fig. 1:**
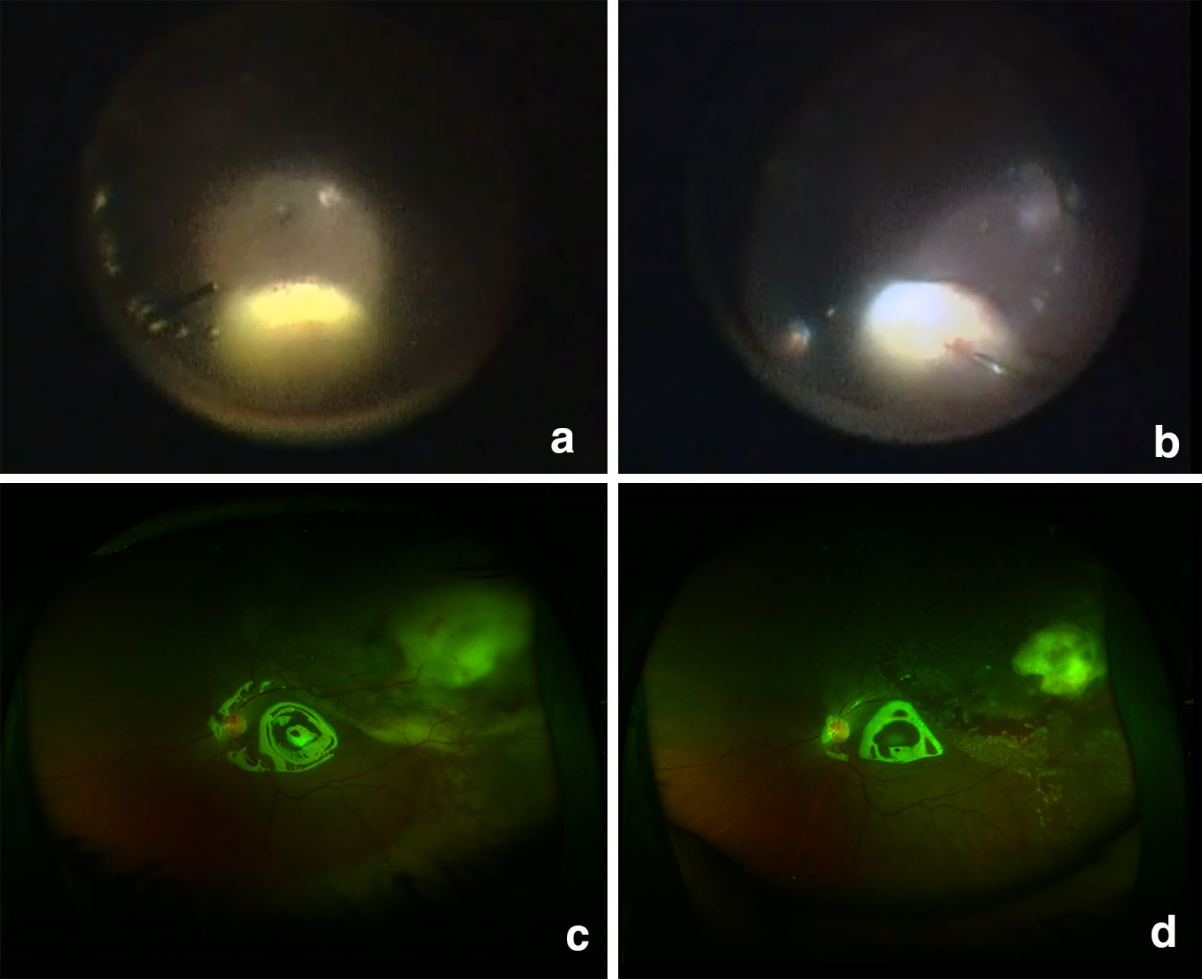
**a** Intraoperative snap shot of subretinal abscess after performing core vitrectomy (preoperative images could not be obtained due to 3+/4+ media haze), **b** intraoperative snapshot showing penetration of the subretinal abscess with 41G needle just before intralesional antibiotic injection, **c** postoperative (day 1) wide angle fundus photograph shows large subretinal abscess, minimal subretinal hemorrhage and significant subretinal exudative fluid surrounding the abscess and tracking inferiorly, **d** postoperative (day 10) fundus photograph shows significant shrinkage of the abscess and resolution of the exudative detachment

Based on the clinical picture, a diagnosis of endogenous endophthalmitis with subretinal abscess was made. She was started on intravenous Vancomycin (40 mg/kg/day) and Ceftriaxone (100 mg/kg/day). Pars plana vitreous biopsy was performed along with injection of intravitreal antibiotics (Vancomycin 1 mg/0.1 ml and Ceftazidime 2.25 mg/0.1 ml). Gram stain and culture of the vitreous specimen did not reveal any organism. Blood culture and urine culture also failed to grow any organism. There was further deterioration of visual acuity and media clarity over the next 24 h along with ultrasound evidence of increase in the size of the abscess. A standard 25G pars plana vitrectomy was hence immediately undertaken. After core vitrectomy and sufficient clearing of media, a yellowish subretinal abscess of approximately 8 × 6 disc areas could be visualized in the superotemporal region (Fig. 1a). Since the abscess was large and had not responded to systemic and intravitreal injections, a decision to administer antibiotics into the lesion was taken. An elevated and relatively less vascular area was identified over the abscess. 41G needle (DORC international, Netherlands) was used to inject Vancomycin (0.05 mg/0.05 ml) directly into the subretinal abscess (Fig. 1b). After air-fluid exchange, silicone oil (1000 cs) was injected to provide internal tamponade.

On the first postoperative day, her best corrected visual acuity (BCVA) was 4/60. There was a track of exudative fluid along the temporal aspect and minimal subretinal bleed adjacent to the abscess (Fig. 1c). On the third postoperative day, there was marginal reduction in the size of the abscess and appreciable decrease in exudative fluid. On day 10, after surgery the subretinal abscess had shrunk significantly and there was absence of any subretinal fluid (Fig. 1d). There was no further increase in subretinal hemorrhage. Visual acuity had improved to 6/24 at this visit and intraocular pressure was normal (13 mmHg). Patient was lost to follow up after this and despite repeated telephonic requests did not report for a follow up due to economic constraints. She is apparently under the care of a local ophthalmologist and continues to maintain 6/24 vision in the involved eye.

## Conclusion

Endogenous endophthalmitis occurs due to septic embolization of the choroid from a metastatic infectious source. Presence of subretinal abscess indicates greater severity of the infection and carries high ocular morbidity and systemic mortality [4, 5]. Hence an aggressive local and systemic therapy is essential for the control of both local disease and systemic condition.

Endogenous endophthalmitis is often seen in patients in whom immunity is significantly diminished. However, immunocompetent individuals with risk factors like intravenous drug abuse, long-term intravenous catheterization, history of gastrointestinal or pelvic surgery, endocarditis, abscess in other parts of the body are also prone to develop endogenous endophthalmitis [6]. Our patient was immunocompetent and there was history of repeated intravenous drug administration.

The most common cause of endogenous endophthalmitis in drug abusers is fungal [7]. Though we could not culture organisms from any of the samples, the rapidity of onset and progression favoured a bacterial etiology. The presence of cellulitis of the leg also hinted towards the possibility of a gram positive organism. Fungal infections have only rarely been implicated as a cause of subretinal abscess. This is said to be because of their propensity to cause vertical invasion across the retina and disseminate into the vitreous cavity [8].

In our case, culture of vitreous, blood and urine remained negative for both bacterial and fungi. It has been reported in the literature that cases of endogenous endophthalmitis presenting as subretinal abscess have low rates of isolation of organisms using aqueous or vitreous biopsy [9, 10]. Subretinal biopsy and aspiration has been shown to yield higher rates of culture positivity in patients with subretinal abscess [11–13]. However, creating retinotomy with conventional techniques in a fragile retina overlying the abscess is known to increase the risk of retinal detachment and proliferative vitreoretinopathy [10]. Due to this reason and the fact that a small (41G) needle was used for intralesional drug delivery in our patient, we did not make an attempt at diagnostic aspiration of the abscess. This could also be considered a limitation in our approach and description of the case report. A single case and relatively short follow up are few other limitations of the report.

No standard management guidelines are available in literature for the treatment of a subretinal abscess because of its rare occurrence and varied clinical presentation. Management options include intravenous antibiotics alone, pars plana vitrectomy with intravitreal antibiotics, pars plana vitrectomy with retinotomy or retinectomy and enucleation. Intravenous route of antibiotic delivery remains the initial modality in the management of endogenous endophthalmitis. Trigui et al. used intravenous ceftriaxone alone in the management of a subretinal abscess caused by Staphylococcus aureus in a diabetic patient with good response [14]. Lakosha et al. had observed incomplete resolution and recurrence of the subretinal abscess alone with systemic trimethoprim-sulfamethoxazole [15].

A poor response to systemic antibiotic therapy results from high choroidal blow and blood retinal barrier which allow only 5 % of the administered drug to actually reach the intended target. Our patient was started on intravenous antibiotics (Vancomycin and Ceftriaxone) to provide empirical cover for common bacterial pathogens known to cause endogenous endophthalmitis. In case of inadequate response to systemic therapy, treatment with intravitreal antibiotics has been suggested to provide high local concentration of the drug.

Pars plana vitrectomy with intravitreal antibiotics has been described as a treatment modality in the management of endogenous endophthalmitis. Tsai et al. have shown adequate response to pars plana vitrectomy with intravitreal injection of Ceftazidime (2 mg/0.1 ml) and Amikacin (0.4 mg/0.1 ml) without retinotomy. They inferred that if the size of the subretinal abscess is smaller than four disc areas, pars plana vitrectomy with intravitreal injection of antibiotics could be successful [16]. However, the abscess was large in size in our case and we felt that pars plana vitrectomy with intravitreal antibiotics alone would not be sufficient to reduce the infectious load within the abscess.

In such cases internal drainage of the contents of subretinal abscess using conventional retinotomy has been reported [10, 12, 13, 17]. However there was a increased incidence of proliferative vitreoretinopathy and retinal detachment in these cases [10, 17]. We concur that the reason for this increased risk is related the large conventional retinotomy and traction induced by active aspiration. To overcome the complications associated with conventional techniques, we opted for a more minimally invasive modality. Use of a 41G macular translocation needle allowed for a minimally invasive means to deliver the drug into the intralesional/subretinal space. We used only one drug as we were doing this procedure for the first time and were not sure how much of the drug we would be able to inject safely into the abscess.

Self-sealing small retinotomy created with a 41G translocation needle can be safely used to deliver drugs in a subretinal lesion decreasing the chance of proliferative vitreoretinopathy and retinal detachment. 41G needle has previously been used as a safe modality for subretinal delivery of bevacizumab, recombinant tissue plasminogen activator and air in the management of submacular bleed associated with wet age related macular degeneration [18].

Subretinal abscess is an extremely rare presentation of endogenous endophthalmitis. Both local and systemic therapies are important to reduce ocular morbidity and systemic morbidity and mortality. A large subretinal abscess may require some form of locally invasive therapy. A 41G translocation needle can be safely used in such cases to create a self-sealing retinotomy to inject intralesional antibiotics.

## Abbreviations

BCVA: best corrected visual acuity
DORC: Dutch Ophthalmic Research Centre
